# Eigenlipids for exploring lipid biology

**DOI:** 10.1016/j.jlr.2026.101060

**Published:** 2026-05-20

**Authors:** Nicholas Sing, Adam Alexander T. Smith, Aleksandar Dakic, Changyu Yi, Agus Salim, Corey Giles, Peter J. Meikle

**Affiliations:** 1 Metabolomics Laboratory, Baker Heart and Diabetes Institute, Melbourne, VIC, Australia; 2 Department of Mathematical and Physical Sciences La Trobe University, Melbourne, VIC, Australia; 3 Department of Cardiovascular Research Translation and Implementation, La Trobe University, Melbourne, VIC, Australia; 4 Friedrich Miescher Institute for Biomedical Research, Basel, Switzerland; 5 Baker Department of Cardiometabolic Health, University of Melbourne, Melbourne, VIC, Australia; 6 Melbourne School of Population and Global Health, University of Melbourne, Melbourne, VIC, Australia; 7 School of Mathematics and Statistics, University of Melbourne, Melbourne, VIC, Australia; 8 Faculty of Medicine, Nursing and Health Sciences, Monash University, Melbourne, VIC, Australia

**Keywords:** Bioinformatics, eigenlipid, eigenmetabolite, lipid modelling, lipid modeling, eigengene, lipidomics, lipids, lipid•, metabolomics

## Abstract

Lipidomics involves the analysis of hundreds or even thousands of lipids within biological systems. Many of these lipids are highly correlated due to similarities in structure or relationships within metabolic pathways. Eigenlipids, as a concept ported over from transcriptomics, can be used to capture these associations and to explore relationships between their constituent lipids and phenotypes of interest. However, gene expression and lipid concentration data differ in how they are structured, requiring additional considerations for eigenlipid analysis. In this review, we discuss how eigenlipids are generated, how they are used to explore lipid metabolism and biology, possible usage pitfalls and finally potential future methodological developments, such as alternative dimensionality reduction methods and generating eigenlipids for predefined lipid sets instead of unsupervised clusters.

The field of lipidomics aims to characterize the entire lipidome of biological systems and study their relationship to observable phenotypes. Thousands of lipid metabolites are essential for living systems, and though lipidomic studies used to be restricted to the analysis of a few hundred metabolites across a limited number of samples, the last decade has seen an explosion in both the depth and breadth of lipids analyzed, as well as in sample sizes, particularly in human population studies. Lipidomics data analysis has thus required the development of new tools to aid in the interpretation of the large numbers of lipids now routinely measured. Eigenlipids are one such tool. Eigenlipids provide a way to summarize groups of highly correlated lipids and thereby analyze phenotypic associations with only a reduced set of summaries. This tool has rapidly gained popularity, and eigenlipid studies now cover a wide variety of human pathologies, as well as other organisms, including plants, microorganisms, and animals (1, 2, 3, 4, 5, 6, 7, 8).

Eigenlipid analysis involves generating low-dimensional representations of clusters of lipids. As first popularized in transcriptomics, the typical first step is to create a correlation-based dissimilarity matrix, which is then used to cluster the lipids. For each cluster (commonly referred to as a "module"), principal component analysis (PCA) is performed. The obtained principal components are ordered by the fraction of total variance they capture, and the first principal component is taken as the module’s eigenlipid representation. This approach summarizes the variations of multiple highly correlated lipids, which are assumed to be biologically related through lipid metabolism. These eigenlipids can then be used to examine associations between lipid clusters and phenotypes.

However, there are potential limitations to the application of eigenlipids generated using this approach. Eigenlipid methodology has largely followed transcriptomic eigengene methodologies. As a result, studies have focused on only the first eigenlipid that represents mostly a single source of variation. This is done by raising the power of the correlation matrix exponent to maximize the tightness of clusters (i.e. a high correlation between lipids within a cluster) (9, 10). This approach may not be fully applicable to lipidomics, where lipids may be synthesized along parallel metabolic pathways or affected by multiple lipid features, such as fatty acyl chain length or headgroup, leading to more complex or interconnected correlation structures than the typical hub model used in transcriptomics.

In this review, we first explore the background and current state of eigenlipids, before considering extensions to typical eigenlipid analysis methodology (Fig. 1). We begin by discussing eigenlipid methodology in terms of its construction and highlight how eigenlipids are used to explore lipid metabolism within biological systems. We then address the limitations of typical approaches and discuss alternative strategies for eigenlipid generation.

**Fig. 1:**
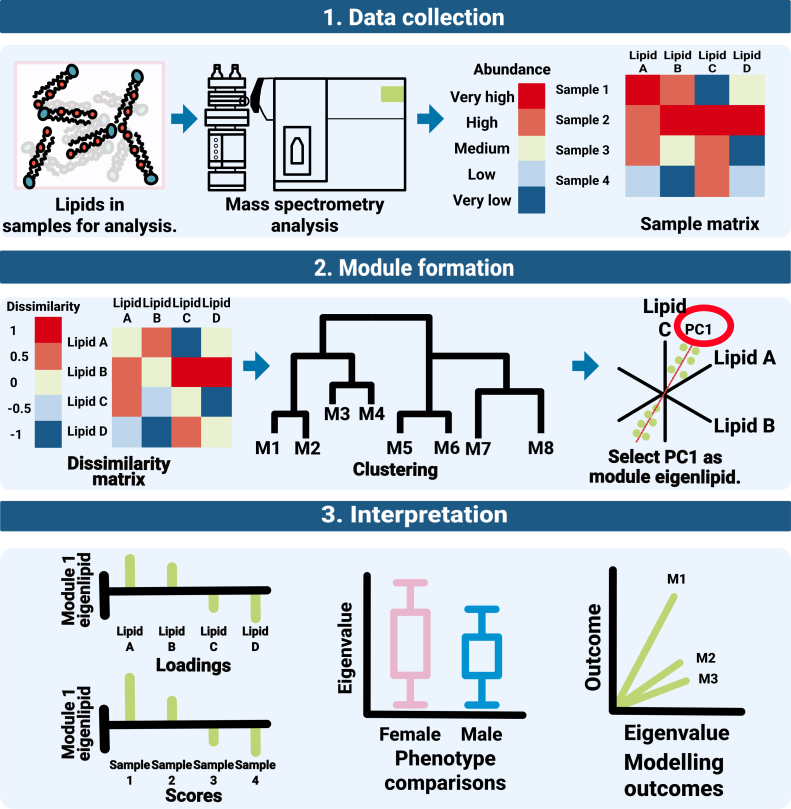
Overview of the creation and application of eigenlipids in lipidomics. (1) Lipid collection provides a data matrix of lipid concentrations; (2) clustering is then performed using a dissimilarity matrix to form module eigenlipids (ME) clusters that are used to then perform PCA; (3) the loadings and scores are then used to analyze eigenlipids with a variety of statistical techniques.

## Eigenlipids in the literature

The transfer of the eigengene concept to lipidomics is a relatively recent phenomenon, with most studies published only in the past three to six years. That said, these studies now cover a wide range of pathologies and organisms, including plants, animals and microorganisms, with varying levels of lipid coverage and study design.

There are several different types of study designs incorporating eigenlipids in the literature. Mishra *et al.* (3), reported a population-based study that modeled the association of eigenlipids with osteoporosis and atherosclerosis, and also compared eigenvalues to understand the effect of disease states on the lipidome of individuals in the cohort. They optimized the dissimilarity matrix for scale-free topology, using methods commonly applied from the Weighted Gene Correlation Network Analysis (WGCNA) R package. Other studies, such as Zhou *et al.* (8) and Serra *et al.* (11), focused on examining relationships within metabolic networks to assess the collective association of lipids along metabolic pathways. In this context, eigenlipids were used to explore the interrelationships between lipids and various diseases. Others, such as Vacy *et al.* (5), focused on identifying hub lipids within clusters of eigenlipids that were highly correlated with disease. Further details can be found in Table 1. With these published eigenlipid applications in mind, we next summarize the main steps to creating and using eigenlipids.

**Table 1:** Examples of eigenlipid studies, pathology examined and type of eigenlipid analysis performed

Study	Pathology	Lipid Coverage	Eigenlipid Analysis	Number of eigenlipids	Cohort Size
Titz *et al.* (1)	Chronic obstructive pulmonary disease	• Glycerolipids • Glycerophospholipids • Sphingolipids • Sterol lipids	• Eigenvalue comparisons.	13	240
Jha *et al.* (2)	Liver	• Glycerolipids • Glycerophospholipids • Sphingolipids	• Eigenvalue comparisons • Linear regression modelling. • Network analysis	8	47 BXD strains containing approximately 5 mice each.
Mishra *et al.* (3)	Osteoperosis	• Glycerolipids • Glycerophospholipids • Sphingolipids • Sterol lipids	• Eigenvalue comparisons • Linear regression modelling. • Multivariate analysis of variance (MANOVA).	12	1,494
Tan *et al.* (4)	Central nervous system, dyslipidaemia	• Glycerolipids • Glycerophospholipids • Sphingolipids • Sterol lipids	• Eigenvalue comparisons	23	4 mice.
Vacy *et al.* (5)	ADHD, ASD	• Glycerolipids, • Glycerophospholipids • Sphingolipids • Sterol lipids	• Correlation analysis • Hub lipid markers. • Network analysis	16	1,074
Fujimara *et al.* (6)	Neonatal gut Microbiome	• Glycerolipids • Glycerophospholipids • Sphingolipids • Sterol lipids	• Linear regression modelling	32	298
Petersen *et al.* (7)	Methods	• Glycerolipids • Glycerophospholipids • Sphingolipids • Sterol lipids	• Linear regression modelling	35	397
Zhou *et al.* (8)	Type-2 Diabetes	• Glycerolipids • Glycerophospholipids • Sphingolipids • Sterol lipids	• Network analysis	7	12 mice
Serra *et al.* (11)	Pear Fruit	• Glycerolipids • Sterol lipids	• Network analysis	10	15 trees
Zhang *et al.* (12)	Beef	• Glycerolipids • Glycerophospholipids • Sphingolipids • Sterol lipids	• Correlation analysis	11	32 chicken samples
Deng *et al.* (13)	Osteoporosis	• Glycerolipids, • Glycerophospholipids • Sphingolipids • Sterol lipids	• Network analysis	36	47
He *et al.* (14)	Preeclampsia	• Glycerolipids • Glycerophospholipids • Sphingolipids • Sterol lipids	• Network analysis • Correlation anlaysis	3	64

## Eigenlipid methodology

Much of the current methodology surrounding eigenlipids stems from earlier studies in the field of transcriptomics. As such, eigenlipids have been derived using the WGCNA R package (15). Typical methodology involves first determining a measure of dissimilarity between lipids, clustering the lipids on this basis (e.g. with hierarchical clustering), and then for each cluster, generating eigenlipids via PCA. These can then be analyzed for associations with phenotypes of interest.

### Dissimilarity matrix

Grouping lipids together requires some measure of which lipids can be considered similar and which ones dissimilar. Such information is typically compiled into a so-called lipid-lipid (otherwise known as interlipid) dissimilarity matrix. The actual measure of dissimilarity between two lipids is often defined as 1 minus the interlipid correlation in the dataset under investigation, typically using Pearson’s correlation, but also Spearman’s or bi-weight mid correlation coefficient has been suggested as a possible alternatives (15).

#### Scale-free topology and hub lipids

In typical eigenlipid analysis, the dissimilarity matrix is raised to an *n*th exponent (denoted β), as a scale-free topology for lipid correlations is commonly assumed. By increasing β in the correlation network, weaker correlations are skewed closer to 0 and higher correlations are skewed closer to 1, favoring hub-like structures. In a scale-free correlation network topology, lipids correlate with a single central hub lipid. Hubs are single lipids found within an eigenlipid cluster that have the highest correlation with all other lipids in the cluster. These lipids are assumed to be the main drivers behind lipid metabolism in each module (5, 10, 16, 17). To determine the β for scale-free topology, scale-independence, and mean connectivity measures are plotted against each other to help manually determine whether the modules generated for each β are too sparse. The plots examine the connectivity of lipids with adjacent lipids (i.e. those least dissimilar according to the dissimilarity matrix) and are fully described in Zhang (18). For these plots, the following power law is assumed for the connectivity of adjacent lipids in a scale-free topology for adjacent lipids:

(1) $$p\left(k\right)∼\;k^{b}$$

*k* is the lipid connectivity of a lipid and is the sum of dissimilarities for adjacent lipids.

β is the exponent used for correlations in the dissimilarity matrix.

p(*k*) is the proportion of lipids that are adjacent in a dissimilarity matrix.

As a result, as p(k) increases, the overall number of connected adjacent lipids is assumed to decrease. Hence, a plot of log(p(k) versus log(k) is plotted for each of the lipids and the lowest value where the R-squared value is ≥ 0.8 is identified as the optimal β for scale-free topology.

To assess connectivity, for each lipid the sum of adjacent dissimilarities with all other lipids is calculated, and the mean of the values of all lipids is calculated as the mean connectivity:

(2) $$\text{Mean}\;\text{connectivity}=\frac{1}{N}\sum k_{i}$$

*N* is the number of lipids in the dissimilarity matrix.

*k*_*i*_ is the *i*th lipid connectivity value.

### Clustering algorithm

#### Generating a dendrogram

With a dissimilarity matrix, we can now group highly correlated lipids together, which can be achieved by any clustering or partitioning algorithm. This is typically done via agglomerative hierarchical clustering using a linkage algorithm, which iteratively merges lipids or clusters, forming a dendrogram. Common linkage algorithms include: single linkage, where the distance between clusters is defined as the minimum distance between any pair of elements between two clusters; average linkage, where the distance between clusters is the average distance between all pairs of elements from each cluster; complete linkage, where the distance between clusters is defined as the maximum distance between any pair of elements between two clusters; and Ward’s method, where the algorithm pairs clusters in order to minimize the within-cluster variance.

#### Cutting the dendrogram into clusters

To obtain different numbers of lipid clusters based on the hierarchical cluster dendrogram, tree-cutting methods can be used. The standard so-called “static” tree-cut approach is to slice the dendrogram at a set dendrogram height (h) or a set number of clusters (k). While simple and generally applicable, these methods can often lead to non-intuitive or biologically irrelevant cluster structures.

To address issues with cluster formation via static tree cutting, Langfelder *et al.* proposed both the dynamic tree cut and dynamic hybrid cut methods (2, 3, 5, 12, 19). These were shown to create more appropriate gene clusters, based on the biological relevance of clusters evaluated using enrichment analysis.

The dynamic tree cut method identifies branch structures in a dendrogram that meet shape (“tightness” within branches, spacing between them, depth of their root, etc.) and minimal size criteria and uses these to define clusters, rather than cutting at a fixed height. The dynamic hybrid cut method additionally considers the original dissimilarity matrix to refine cluster allocation, resulting in clusters more robust to unclear dendrogram structures and noisy data.

K-means clustering sometimes features in eigenlipid studies as an alternative to hierarchical clustering (20). This methodology starts by assigning lipids to one of k clusters, typically at random or via a simple clustering approach. Then, the centroid of each cluster is calculated, and lipids are assigned to their closest centroid. This process iterates until a convergence criteria is met. However, this form of clustering is substantially less common, as most studies utilize the WGCNA R package, which provides functions for generating eigenlipids from hierarchical clusters.

### Generating and using eigenlipids

#### Dimensionality reduction

To generate eigenlipids, dimensionality reduction, typically via PCA, is performed for each cluster. Commonly, the first principal component (which captures the most variation) is selected as the eigenlipid representation for each cluster. As the exponent (β) of the dissimilarity matrix used for cluster generation is typically raised (15), variation is generally already concentrated into the first principal component.

#### Using enrichment analysis to identify the relationship of eigenlipid modules with lipid biology

The eigenlipid approaches outlined above are unsupervised, in that lipids are clustered together based solely on the data, and not on any prior knowledge. This allows for the discovery of new associations but also means that lipids are not guaranteed to cluster in line with known lipid biology. More problematically for clinical studies, lipid clusters are also highly likely to change between studies and cohorts. A tool enabling the biological interpretation of the contents of eigenlipid clusters is enrichment analysis (21). This method relies on lipids being annotated with terms from a controlled vocabulary (or more generally an ontology) and statistically tests for the over- (or more rarely under-) representation of these terms in lipid clusters. This approach has been extensively used in lipidomics, and there are several potential databases that exist in the field that make it possible to utilize the same form of analysis for eigenlipids (22, 23, 24, 25, 26, 27, 28, 29).

#### Exploring lipid metabolism with eigenlipids

The principal component scores for the generated eigenlipids can be used to investigate the biological relationships of eigenlipid modules with phenotypes of interest. This can include exploring the distribution of eigenlipid scores (1, 2, 4, 16, 30, 31, 32) in relation to specific traits such as states of disease and metabolic profiles, or alternatively using some form of regression modeling/testing, such as linear regression, t-testing or analysis of variance (ANOVA) (1, 2, 4, 16, 30, 31, 32). Furthermore, network analysis methods can also be used to explore the associations between different eigenlipid modules and identify the roles lipid clusters play in metabolic pathways (21, 31). This may uncover insights into lipid co-regulation and interdependencies related to different interrelated or overlapping pathways in lipid metabolism.

#### Comparison of eigenlipid methodology with traditional lipidomic data analysis

Traditional lipidomic data analysis relies on a variety of univariate and multivariate approaches. Univariate approaches, such as t-tests (33), ANOVA (33), linear/logistic regression (34), as well as non-parametric tests, allow researchers to assess sample differences on a per lipid basis. While highly interpretable, testing hundreds to thousands of lipid species introduces a severe multiple testing burden, requiring stringent *P*-value corrections that ultimately reduce statistical power (35).

In addition to univariate statistical testing, lipidomics researchers have also utilized unsupervised techniques and dimensionality reduction, such as clustering and PCA. Clustering, such as hierarchical and k-means, has been used to create groups of lipids based on correlation/similarity (36, 37). However, a primary limitation of standard clustering is that it does not intrinsically generate a robust, unified summary metric for each group. In contrast, dimensionality reduction methods, such as PCA, have been used to summarize the major axes of variation across an entire dataset (38). While the global PCA effectively decomposes the major sources of variation, it can provide a challenge for interpretation or obscure the effects of small groups of biologically distinct sub-groups.

The eigenlipid methodology addresses these limitations by combining the biological specificity of clustering with the mathematical efficiency of PCA. First, highly correlated lipids are clustered into distinct modules. Subsequently, PCA is applied within each module to extract the first principal component, which serves as a single quantitative summary of that module—the module eigenlipid. By using this eigenlipid score in subsequent statistical testing, researchers can evaluate the collective effects of co-regulated lipid networks, effectively capture systems-level biological changes while drastically mitigating the multiple testing burden.

## Potential drawbacks of eigenlipid methodology

There are some key drawbacks of eigenlipid methodology. These include: (1) eigenlipids are typically generated from unsupervised clusters, (2) tools that are commonly used as part of eigenlipid generation assume an inherent scale-free topology and (3) dimensionality reduction is performed via PCA. The drawbacks of each of these aspects is discussed below.

### Unsupervised clustering

As pointed out above, eigenlipids are generated using unsupervised clustering, which makes their interpretation non-trivial and limits their transferability to other datasets. While it may be possible to use an eigenlipid in another cohort for model prediction, this method may not account for differences in lipid metabolism between the two cohorts.

### Lack of scale-free topology

There are many assumptions in methods used for the analysis of gene expression datasets that do not apply to the examination of lipids in metabolic pathways (39). Most studies assume that scale-free topology holds true for lipids. Whilst this may be true of transcriptomic datasets, lipidomic datasets are unlikely to present the same correlation structures, due to (1) the existence of simultaneously occurring parallel pathways, (2) more complex biology due to lipids being composed of multiple parts, such as headgroup and side chains, and (3) there is the risk that even if hub lipids exist, they can be low abundance species, weakening correlations due to noise and detection limits.

### Limitations of principal component analysis

Compared to other dimensionality reduction methods, the PCA method is relatively simple to interpret and has a lower computational time. However, there are limitations of the PCA approach to dimensionality reduction: (1) the first principal component represents only the largest source of linear variation in a cluster, and (2) PCA is a linear dimensionality reduction method and works best with Gaussian data.

Lipids exhibit diverse structural features, such as head groups, side chains, and functional groups that derive from different metabolic pathways. This can lead to multiple sources of variation present in an eigenlipid cluster. In an eigenlipid analysis using a basic dissimilarity metric, this would lead to multiple sources of variation in an eigenlipid cluster that cannot be completely captured by the first principal component, forcing the selection of multiple eigenlipids per cluster. However, in typical eigenlipid analysis, the dissimilarly matrix features a high β, which biases clusters to be small, with much of their variation represented by only the first principal component, and later components being mostly noise. Care must thus be exercised when selecting the β and selecting how many clusters and eigenlipids within to consider for further analysis, and choices will be objective-dependent.

Lipidomics datasets are often non-normally distributed and right-skewed (40). Commonly, these features are mitigated via logarithmic transformation of lipid concentrations; however, this does not completely lead to fully Gaussian data (41). Additionally, there are also many nonlinear effects in metabolism that can affect lipid abundance, including enzyme kinetics and gene regulatory effects (42). These will all limit the interpretability and biological relevance of the resulting principal components derived from the data.

### Robustness and reproducibility of eigenlipids

Traditional approaches for eigenlipids generation consist of unsupervised methods, which will always generate an output regardless of whether they have biological significance or not. Therefore, care must be taken to avoid data leakage, which could lead to overfitting eigenlipid modules with poor biological significance. Such overfitted eigenlipids will not be replicable across different datasets, and model estimates cannot be transferred between studies. Where multiple datasets are available, or eigenlipids can be generated from different tissues, there are statistical approaches to evaluate the reproducibility of the modules, for instance, with WGCNA (43), NetRep (44) or enrichment analysis to compare eigenlipid enrichments (21). Where sample variability or outliers may be a concern, techniques such as bootstrapping PCA can be used to measure the stability of eigenlipids (45, 46).

## Extensions of eigenlipid methodology

The limitations of the standard eigenlipid approach can be addressed by variations to the methodology, including: (1) generating supervised eigenlipids in place of unsupervised eigenlipids, (2) using lower-order principal components, and (3) alternative dimensionality reduction methods.

### Supervised eigenlipids

Supervised eigenlipids are generated for biologically relevant, pre-defined lipid clusters, established based on pathways featured in a lipid ontology. This can be performed with ontologies such as those used in enrichment analyses. Clusters can be formed via the grouping of lipids based on class, subclass, structure, side chains, and association along known metabolic pathways. This has recently been attempted for broader metabolomic studies, which include lipids in modules based on pathways in the KEGG pathway database and a selection of glycerolipid modules largely discriminated by lipid class or combinations of different classes (2). This approach also has advantages for using the same lipids in multiple eigenlipid modules. For example, Ja *et al.* (2), examined the interrelationship between triacylglycerol and phospholipid species in liver metabolism that were not ordinarily observable when they examined standalone triacylglycerol and phospholipid eigenlipid modules.

### Lower order principal component eigenlipids

Lower order principal components are routinely analyzed for entire data matrices in lipidomics. This includes many of the same regression modelling, analysis of traits (47, 48). However, eigenlipid analysis using lower order principal components for clustered lipids is yet to be explored. If a scale-free topology is not assumed, then β can be reduced to create clusters with more sources of variation that can be analyzed via lower order principal components. Each eigenlipid principal component will then capture different sources of biological variation and can be interpreted separately.

### Alternative eigenlipid generation methods

While PCA appears to be the extremely common approach to eigenlipid analysis in wider systems biology, it is by no means the only one. Other possible dimensionality reduction methods include partial least squares (PLS) regression, sparse-PCA/PLS, independent component analysis (ICA), kernel PCA (KPCA) and variational autoencoders (VAE). In the past, these methods have been performed on the entire lipid data matrix rather than for clustered lipids. For many of these approaches, it should be noted compute time can be much longer.

#### Partial least squares regression

PLS regression is a supervised dimensionality reduction method that can be used to generate eigenlipids that are specific to an outcome that is being modeled. PLS regression focuses on finding the linear combination of lipids that has the maximum covariance with the outcome. Eigenlipids generated via PLS can potentially be more highly correlated with an outcome than PCA, where the outcome is not considered, which may be useful for increasing power when examining the associations of eigenlipids with biology. However, a downside to this approach is that the covariance between outcome and lipids may lead to overfitting, as PLS is trained on the association between lipids and outcomes and not unknown or alternative sources of variation (49). Although there are mitigation strategies that can be employed, such as cross-validation and regularization.

#### Sparse principal component analysis and sparse-partial least squares

Standard PCA and PLS generate components based on linear combinations of all features, making interpretation non-trivial. Sparse approaches alleviate this by restricting the number of features used to generate the component linear combinations. Several different forms of sparse-PCA and sparse-PLS approaches exist, which commonly involve applying an “elastic net” or “lasso” penalty parameter to loadings estimated for the latent variables (such as principal components) (50, 51, 52). This pushes the less relevant loadings to 0, decreasing the number of lipids contributing to each latent variable, and thereby increasing its interpretability. In clusters, many of the lipids may be of similar class or have similar associations along metabolic pathways and share collinearity. By using sparse approaches to PCA, the effects of these lipids can be reduced to only the key drivers of variation in a cluster.

#### Independent component analysis for non-Gaussian dimensionality reductions

Another alternative approach to PCA is to utilize ICA-based methods, which can identify non-Gaussian-distributed factors. ICA produces components (termed independent components) that are highly sparse, maximally independent (i.e. beyond simple uncorrelatedness), and compared with PCA, show little overlap with each other in terms of gene/lipid/metabolite content. ICA has been previously used by Krumsiek *et al.* in a metabolomics study, which included the analysis of a variety of lipids. The independent components were more strongly associated with outcomes than principal components generated from PCA and were more consistently associated with sub-pathways of lipid metabolism.

#### Kernel principal component analysis and autoencoders for non-linear non-Gaussian dimensionality reduction

To identify non-linear and non-Gaussian variation among lipids, two possible dimensionality reduction methods are Kernel Principal Component Analysis (KPCA) and Variational Autoencoders (VAE). KPCA applies a non-linear kernel mapping function to the lipid data matrix (53). However, the kernel must be appropriately selected to fit the data. In contrast, VAE is a dimensionality reduction technique in which latent variables correspond to hidden nodes in a neural network summarizing the variation in high-dimensional data (54).

Gomari *et al.* (55) recently compared PCA, KPCA, and VAE in evaluating the association of metabolites, including lipids with type 2 diabetes, acute myeloid leukemia, and schizophrenia. KPCA and VAE outperformed PCA for non-linear data, with VAE showing the strongest associations and generalizability across cohorts. Notably, VAE captured a broader spectrum of lipid features, including varied head groups, that were consistent across diseases. In type 2 diabetes, the most associated VAE dimension reflected contributions from lysolipids, n-3 and n-6 polyunsaturated lipids, and branched-chain fatty acids. This diversity was not observed with PCA or KPCA. Furthermore, this was generalizable across cohorts. However, despite the methods' apparent generalizability, VAE typically requires regularization to mitigate overfitting, which is a common concern with this approach (56).

## Conclusion

There are now a wide variety of studies utilizing eigenlipids to explore the role of lipids within biological systems, and their associations with outcomes of interest. However, typical eigenlipid analysis inherits assumptions and limitations from transcriptomics, where correlations are sparser, and gene clusters often have a hub gene, unlike the clusters of dense sets of highly correlated lipids. Studies mostly focus on first principal component eigenlipids, which may miss variation in lower-order components and assume linearity and normality. There is potential to use more atypical but hopefully more biologically relevant methodology in eigenlipid analysis, such as supervised eigenlipids, lower-order components, or alternative dimensionality reduction methods such as sparse-PCA/PLS, ICA, KPCA and VAEs. However, caution must be exercised with these approaches there are issues with overfitting and correct parameter selection.

## Conflict of interest

The authors declare that they have no conflicts of interest with the contents of this article.

## References

[1] Titz B., Luettich K., Leroy P., Boue S., Vuillaume G., Vihervaara T. (2016). Alterations in serum polyunsaturated Fatty acids and eicosanoids in patients with mild to moderate Chronic Obstructive Pulmonary Disease (COPD). Int. J. Mol. Sci..

[2] Jha P., McDevitt M.T., Gupta R., Quiros P.M., Williams E.G., Gariani K. (2018). Systems analyses reveal physiological roles and genetic regulators of liver lipid species. Cell Syst..

[3] Mishra B.H., Mishra P.P., Mononen N., Hilvo M., Sievänen H., Juonala M. (2020). Lipidomic architecture shared by subclinical markers of osteoporosis and atherosclerosis: the Cardiovascular Risk in Young Finns Study. Bone.

[4] Tan D., Konduri S., Erikci Ertunc M., Zhang P., Wang J., Chang T. (2023). A class of anti-inflammatory lipids decrease with aging in the central nervous system. Nat. Chem. Biol..

[5] Vacy K., Thomson S., Moore A., Eisner A., Tanner S., Pham C. (2024). Cord blood lipid correlation network profiles are associated with subsequent attention-deficit/hyperactivity disorder and autism spectrum disorder symptoms at 2 years: a prospective birth cohort study. EBioMedicine.

[6] Fujimura K.E., Sitarik A.R., Havstad S., Lin D.L., Levan S., Fadrosh D. (2016). Neonatal gut microbiota associates with childhood multisensitized atopy and T cell differentiation. Nat. Med..

[7] Pedersen H.K., Forslund S.K., Gudmundsdottir V., Petersen A.Ø., Hildebrand F., Hyötyläinen T. (2018). A computational framework to integrate high-throughput ‘-omics’ datasets for the identification of potential mechanistic links. Nat. Protoc..

[8] Zhou Z., Liu J., Liu J. (2024). Application of weighted gene Co-Expression network analysis to metabolomic data from an ApoA-I knockout mouse model. Molecules.

[9] Huneault H.E., Chen C.-Y., Cohen C.C., Liu X., Jarrell Z.R., He Z. (2024). Lipidome changes associated with a diet-induced reduction in hepatic fat among adolescent boys with metabolic dysfunction-associated steatotic liver disease. Metabolites.

[10] Li H., Ma Y., Feng N., Wang W., He C. (2022). Exploration of potential biomarkers for type 2 diabetes by UPLC-QTOF-MS and WGCNA of skin surface lipids. Clin. Cosmet. Invest. Dermatol..

[11] Serra S., Sullivan N., Mattheis J.P., Musacchi S., Rudell D.R. (2018). Canopy attachment position influences metabolism and peel constituency of European pear fruit. BMC Plant Biol..

[12] Zhang T., Ding H., Chen L., Zhang S., Wu P., Xie K. (2021). Characterization of chilled chicken spoilage using an integrated microbiome and metabolomics analysis. Food Res. Int..

[13] Deng D., Pan C., Wu Z., Sun Y., Liu C., Xiang H. (2021). An integrated metabolomic study of osteoporosis: discovery and quantification of hyocholic acids as candidate markers. Front. Pharmacol..

[14] He B., Liu Y., Maurya M.R., Benny P., Lassiter C., Li H. (2021). The maternal blood lipidome is indicative of the pathogenesis of severe preeclampsia. J. Lipid Res..

[15] Langfelder P., Horvath S. (2008). WGCNA: an R package for weighted correlation network analysis. BMC Bioinform..

[16] Xu J., Bankov G., Kim M., Wretlind A., Lord J., Green R. (2020). Integrated lipidomics and proteomics network analysis highlights lipid and immunity pathways associated with Alzheimer's disease. Transl. Neurodegener..

[17] Zeng W., Beyene H.B., Kuokkanen M., Miao G., Magliano D.J., Umans J.G. (2022). Lipidomic profiling in the strong heart study identified American Indians at risk of chronic kidney disease. Kidney Int..

[18] Zhang B., Horvath S. (2005). A general framework for weighted gene Co-Expression network analysis journal statistical applications. Genet. Mol. Biol..

[19] Langfelder P., Zhang B., Horvath S. (2007). Defining clusters from a hierarchical cluster tree: the dynamic tree cut package for R. Bioinformatics.

[20] Mares J., Costa A.P., Dartora W.J., Wartchow K.M., Lazarian A., Bennett D.A. (2024). Brain and serum lipidomic profiles implicate lands cycle acyl chain remodeling association with APOEε4 and mild cognitive impairment. Front. Aging Neurosci..

[21] Langfelder P., Horvath S. (2007). Eigengene networks for studying the relationships between co-expression modules. BMC Syst. Biol..

[22] Wishart D.S., Guo A., Oler E., Wang F., Anjum A., Peters H. (2022). Hmdb 5.0: the Human Metabolome Database for 2022. Nucleic Acids Res..

[23] Fahy E., Subramaniam S., Murphy R.C., Nishijima M., Raetz C.R.H., Shimizu T. (2009). Update of the LIPID MAPS comprehensive classification system for lipids. J. Lipid Res..

[24] Aimo L., Liechti R., Hyka-Nouspikel N., Niknejad A., Gleizes A., Götz L. (2015). The SwissLipids knowledgebase for lipid biology. Bioinformatics.

[25] Molenaar M.R., Jeucken A., Wassenaar T.A., van de Lest C.H.A., Brouwers J.F., Helms J.B. (2019). LION/web: a web-based ontology enrichment tool for lipidomic data analysis. GigaScience.

[26] Acevedo A., Durán C., Ciucci S., Gerl M., Cannistraci C.V. (2018). LIPEA: lipid pathway enrichment analysis. bioRxiv.

[27] Consortium T.U. (2018). UniProt: a worldwide hub of protein knowledge. Nucleic Acids Res..

[28] Kanehisa M., Furumichi M., Sato Y., Ishiguro-Watanabe M., Tanabe M. (2021). KEGG: integrating viruses and cellular organisms. Nucleic Acids Res..

[29] Fabregat A., Jupe S., Matthews L., Sidiropoulos K., Gillespie M., Garapati P. (2017). The reactome pathway knowledgebase. Nucleic Acids Res..

[30] Clària J., Curto A., Moreau R., Colsch B., López-Vicario C., Lozano J.J. (2021). Untargeted lipidomics uncovers lipid signatures that distinguish severe from moderate forms of acutely decompensated cirrhosis. J. Hepatol..

[31] Gonzales G.B., Brals D., Sonko B., Sosseh F., Prentice A.M., Moore S.E. (2021). Plasma lipids and growth faltering: a longitudinal cohort study in rural Gambian children. Sci. Adv..

[32] Djekic D., Shi L., Calais F., Carlsson F., Landberg R., Hyötyläinen T. (2020). Effects of a lacto-ovo-vegetarian diet on the plasma lipidome and its Association with atherosclerotic burden in patients with coronary artery Disease-A randomized, open-label, cross-over study. Nutrients.

[33] Eghlimi R., Shi X., Hrovat J., Xi B., Gu H. (2020). Triple negative breast cancer detection using LC–MS/MS lipidomic profiling. J. Proteome Res..

[34] Rauschert S., Uhl O., Koletzko B., Kirchberg F., Mori T.A., Huang R.-C. (2016). Lipidomics reveals associations of phospholipids with obesity and insulin resistance in young adults. J. Clin. Endocrinol. Metab..

[35] Giles C., Takechi R., Lam V., Dhaliwal S.S., Mamo J.C.L. (2018). Contemporary lipidomic analytics: opportunities and pitfalls. Prog. Lipid Res..

[36] Draisma H.H.M., Reijmers T.H., Meulman J.J., van der Greef J., Hankemeier T., Boomsma D.I. (2013). Hierarchical clustering analysis of blood plasma lipidomics profiles from mono- and dizygotic twin families. Eur. J. Hum. Genet..

[37] Aramaki S., Tsuge S., Islam A., Eto F., Sakamoto T., Oyama S. (2023). Lipidomics-based tissue heterogeneity in specimens of luminal breast cancer revealed by clustering analysis of mass spectrometry imaging: a preliminary study. PLoS One.

[38] Zhou X., Mao J., Ai J., Deng Y., Roth M.R., Pound C. (2012). Identification of plasma lipid biomarkers for prostate cancer by lipidomics and bioinformatics. PLoS One.

[39] Lee K.S., Su X., Huan T. (2025). Metabolites are not genes — avoiding the misuse of pathway analysis in metabolomics. Nat. Metab..

[40] Mundra P.A., Shaw J.E., Meikle P.J. (2016). Lipidomic analyses in epidemiology. Int. J. Epidemiol..

[41] Krumsiek J., Suhre K., Illig T., Adamski J., Theis F.J. (2011). Gaussian graphical modeling reconstructs pathway reactions from high-throughput metabolomics data. BMC Syst. Biol..

[42] Schwahn K., Beleggia R., Omranian N., Nikoloski Z. (2017). Stoichiometric correlation analysis: principles of metabolic functionality from Metabolomics data. Front. Plant Sci..

[43] Langfelder P., Luo R., Oldham M.C., Horvath S. (2011). Is my network module preserved and reproducible?. PLoS Comput. Biol..

[44] Ritchie S.C., Watts S., Fearnley L.G., Holt K.E., Abraham G., Inouye M. (2016). A scalable permutation approach reveals replication and preservation patterns of network modules in large datasets. Cell Syst..

[45] Fisher A., Caffo B., Schwartz B., Zipunnikov V. (2016). Fast, exact bootstrap principal component analysis for p > 1 million. J. Am. Stat. Assoc..

[46] Shannon C.P., Chen V., Takhar M., Hollander Z., Balshaw R., McManus B.M. (2016). SABRE: a method for assessing the stability of gene modules in complex tissues and subject populations. BMC Bioinform..

[47] Chen Y., Li K., Zhao H., Hao Z., Yang Y., Gao M. (2022). Integrated lipidomics and network pharmacology analysis to reveal the mechanisms of berberine in the treatment of hyperlipidemia. J. Transl. Med..

[48] Wu X., Zhu J.-C., Zhang Y., Li W.-M., Rong X.-L., Feng Y.-F. (2016). Lipidomics study of plasma phospholipid metabolism in early type 2 diabetes rats with ancient prescription huang-qi-san intervention by UPLC/Q-TOF-MS and correlation coefficient. Chem. Biol. Interact..

[49] Deng B.-C., Yun Y.-H., Liang Y.-Z., Cao D.-S., Xu Q.-S., Yi L.-Z. (2015). A new strategy to prevent over-fitting in partial least squares models based on model population analysis. Analytica Chim. Acta.

[50] Cai T.T., Ma Z., Wu Y. (2013). Sparse PCA: optimal rates and adaptive estimation. Ann. Stat..

[51] Chun H., Keleş S. (2010). Sparse partial least squares regression for simultaneous dimension reduction and variable selection. J. R. Stat. Soc. Ser. B Stat. Methodol..

[52] Lê Cao K.-A., Boitard S., Besse P. (2011). Sparse PLS discriminant analysis: biologically relevant feature selection and graphical displays for multiclass problems. BMC Bioinform..

[53] Schölkopf B., Smola A., Müller K.-R. (1997).

[54] Hinton G.E., Salakhutdinov R.R. (2006). Reducing the dimensionality of data with neural networks. Science.

[55] Gomari D.P., Schweickart A., Cerchietti L., Paietta E., Fernandez H., Al-Amin H. (2022). Variational autoencoders learn transferrable representations of metabolomics data. Commun. Biol..

[56] Takida Y., Liao W.-H., Lai C.-H., Uesaka T., Takahashi S., Mitsufuji Y. (2022). Preventing oversmoothing in VAE via generalized variance parameterization. Neurocomputing.

